# Engineering allosteric inhibition of homoserine dehydrogenase by semi-rational saturation mutagenesis screening

**DOI:** 10.3389/fbioe.2023.1336215

**Published:** 2024-01-03

**Authors:** Xinyang Liu, Jiao Liu, Zhemin Liu, Qianqian Qiao, Xiaomeng Ni, Jinxing Yang, Guannan Sun, Fanghe Li, Wenjuan Zhou, Xuan Guo, Jiuzhou Chen, Shiru Jia, Yu Zheng, Ping Zheng, Jibin Sun

**Affiliations:** ^1^ College of Biotechnology, Tianjin University of Science and Technology, Tianjin, China; ^2^ Key Laboratory of Engineering Biology for Low-carbon Manufacturing, Tianjin Institute of Industrial Biotechnology, Chinese Academy of Sciences, Tianjin, China; ^3^ National Technology Innovation Center of Synthetic Biology, Tianjin, China; ^4^ State Key Laboratory of Food Nutrition and Safety, Tianjin University of Science and Technology, Tianjin, China

**Keywords:** homoserine dehydrogenase, allosteric inhibition, semi-rational design, high-throughput screening (HTS), *Corynebacterium glutamicum*

## Abstract

Allosteric regulation by pathway products plays a vital role in amino acid metabolism. Homoserine dehydrogenase (HSD), the key enzyme for the biosynthesis of various aspartate family amino acids, is subject to feedback inhibition by l-threonine and l-isoleucine. The desensitized mutants with the potential for amino acid production remain limited. Herein, a semi-rational approach was proposed to relieve the feedback inhibition. HSD from *Corynebacterium glutamicum* (*Cg*HSD) was first characterized as a homotetramer, and nine conservative sites at the tetramer interface were selected for saturation mutagenesis by structural simulations and sequence analysis. Then, we established a high-throughput screening (HTS) method based on resistance to l-threonine analog and successfully acquired two dominant mutants (I397V and A384D). Compared with the best-ever reported desensitized mutant G378E, both new mutants qualified the engineered strains with higher production of *Cg*HSD-dependent amino acids. The mutant and wild-type enzymes were purified and assessed in the presence or absence of inhibitors. Both purified mutants maintained >90% activity with 10 mM l-threonine or 25 mM l-isoleucine. Moreover, they showed >50% higher specific activities than G378E without inhibitors. This work provides two competitive alternatives for constructing cell factories of *Cg*HSD-related amino acids and derivatives. Moreover, the proposed approach can be applied to engineering other allosteric enzymes in the amino acid synthesis pathway.

## 1 Introduction

Allosteric inhibition is ubiquitous in the biosynthesis pathways of amino acids, which was evolved to effectively modulate the metabolism for cell growth and survival under different nutritional environments (Leander et al., 2020). When sufficient amino acids exist for cell growth, they may feedback inhibit the corresponding key enzymes involved in biosynthesis since the amino acids are usually the allosteric inhibitors of the enzymes (Sander et al., 2019). Therefore, removing allosteric feedback inhibition is imperatively demanded to achieve superior amino acid production. The traditional method for relieving feedback inhibition is mainly based on whole-cell mutagenesis (chemical or physical) and screening amino acid analog-resistant mutants (Sahm et al., 1996). Recently, computer-aided rational design strategies based on the amino acid sequence or protein structure have been successfully applied to relieve feedback inhibition (Chen et al., 2011a; Geng et al., 2013). For example, rational mutations of aspartokinase, the first rate-limiting enzyme for the biosynthesis of aspartate family amino acids, have been designed by co-evolutionary analysis or in combination with molecular dynamics to successfully relieve the feedback inhibition in both amino acid-producing chassis of *Escherichia coli* and *Corynebacterium glutamicum* (Chen et al., 2011a; Chen et al., 2011b). However, obtaining desensitized mutants with high enzyme activities is still tricky solely through rational design strategies, especially for oligomeric enzymes such as homoserine dehydrogenase. Thus, the semi-rational approach combining a sensible selection of mutation sites and intelligent mutant library screening was usually considered for engineering key enzymes (Liu et al., 2022).

Homoserine dehydrogenase (HSD) is a key enzyme in the aspartate pathway for the biosynthesis of various amino acids and derivatives (Supplementary Figure S1), such as l-threonine, l-isoleucine, l-methionine, and l-homoserine (Li et al., 2017). The crystal structures of HSDs from several microbes have been published, and the catalytic mechanism was revealed, which exists as a dimer or a tetramer (Navratna et al., 2015; Akai et al., 2019). The HSD of *C. glutamicum* (*Cg*HSD, encoded by the *hom* gene) is subjected to strong feedback inhibition by l-threonine and weak feedback inhibition by l-isoleucine. Although the crystal structure of *Cg*HSD has not been reported, potential l-threonine binding sites at *Cg*HSD have been predicted, and many site-directed mutations were rationally designed to deregulate the allosteric inhibition (Chen et al., 2015). These mutants (such as D375A and L380E) efficiently removed feedback inhibition; unfortunately, less than 10% of the specific activity of the wild-type enzyme remained even in the absence of the inhibitors, which hinders the application in amino acid production. Only one feedback-resistant mutant (G378E), identified from an l-threonine-producing strain generated by classical mutagenesis breeding, exhibits good performance for developing efficient amino acid producers (Sahm et al., 1996). More inhibition-relieved mutants with high enzyme activity are eagerly required, which will not only provide preferable mutants for cell factory construction but also benefit the understanding of the allosteric regulatory mechanism of *Cg*HSD.

In this study, the oligomeric state of *Cg*HSD was confirmed as a homotetramer by size-exclusion chromatography (SEC). Homology modeling and docking studies were carried out. The conservative amino acid residues at the tetramer interface within the effector binding domain were then selected to construct saturated mutant libraries. Two new mutants (A384D and I397V) were successfully screened by a high-throughput screening (HTS) method, which efficiently relieved the feedback inhibition and bore high enzymatic activities simultaneously. This study provides *Cg*HSD mutants beneficial for aspartate family amino acid production.

## 2 Materials and methods

### 2.1 Strains and culture conditions

The strains used in this study are listed in Supplementary Table S1. *E. coli* Trans1-T1 (Transgen, China) used for plasmid cloning, and *E. coli* Transetta (DE3) (Transgen, China) used for protein expression were grown at 37°C and 220 rpm in Luria–Bertani (LB) medium. *C. glutamicum* was grown at 30°C and 220 rpm in TSB (pH 7.2), which contains 5 g/L glucose, 9 g/L soya peptone, 5 g/L yeast extract, 1 g/L K_2_HPO_4_·3H_2_O, 0.1 g/L MgSO_4_·7H_2_O, 3 g/L urea, 0.5 g/L succinic acid, 10 μg/L biotin, 100 μg/L vitamin B1 and 20 g/L MOPS.

MM medium (pH 7.2) contains 5 g/L glucose, 1 g/L KH_2_PO_4_, 5 g/L (NH_4_)_2_SO_4_, 0.4 g/L MgSO_4_·7H_2_O, 0.5 g/L NaCl, 2 g/L urea, 200 μg/L biotin, 100 μg/L vitamin B1, 100 μg/L vitamin B5, 0.03 g/L vitamin B3, 0.09 mg/L Na_2_B_4_O_7_·10H_2_O, 0.04 mg/L (NH_4_)_6_Mo_7_O_24_·4H_2_O, 0.01 mg/L ZnSO_4_·7H_2_O, 0.01 mg/L CuSO_4_·5H_2_O, 0.01 mg/L MnCl_2_·4H_2_O, 1 mg/L FeCl_3_·6H_2_O, 0.01 mg/L CaCl_2_. Kanamycin (50 μg/mL) or chloramphenicol (20 μg/mL) was added to the medium when *E. coli* was cultivated. For *C. glutamicum* and its mutants, kanamycin (25 μg/mL) was added to the medium.

### 2.2 Construction of plasmids and strains

Plasmids used in this study are listed in Supplementary Table S2. The primers of constructing plasmids and details are listed in Supplementary Table S3. Services of primer synthesis and DNA sequencing were provided by GENEWIZ Inc. (China). Plasmids were constructed via recombination, which was conducted using the ClonExpress MultiS One-Step Cloning Kit (Vazyme, China).

To construct *hom* deletion plasmid pCas9-gRNA-Δ*hom*, five fragments were amplified and ligated, which contain two pieces of Cas9, a targeting gRNA (5′- AAC​ACG​GGT​GTG​GAA​AGC​GA -3′) designed by sgRNAcas9 tool (Xie et al., 2014) and two HR arms. To construct the plasmid pEC-*hom*, the *hom* gene was amplified from *C. glutamicum* ATCC 13032, and the backbone was amplified from pEC-XK99E. The two fragments were ligated via recombination. For *hom*-*thrB* operon overexpression, the *thrB* gene was amplified from the genome of *C*. *glutamicum,* and the plasmid pEC- *homthrB* was constructed based on pEC-*hom.* To construct the plasmid pEC-*homTLA*, the fragment of *lysC* ^I293Y^-*asd* was amplified from the genomic DNA of ZCgLJ6 and was inserted into plasmid pEC-*homthrB*. The mutations were introduced by mutagenic primers. To construct the pET-*hom* and its derived plasmids, the backbone was amplified from pET-28a.The *hom* gene was amplified from the genomic DNA of *C. glutamicum* ATCC 13032, and the mutagenic primers were used to introduce the mutations.

Electroporation of *C. glutamicum* was performed as described previously (Ruan et al., 2015). To construct *hom* deletion strain LCgL1, plasmid pCas9-gRNA-*Δhom* was transformed into ZCgLJ6 by electroporation. The *hom* gene was deleted as described (Liu et al., 2017). Plasmids constructed based on pEC-XK99E were transformed into LCgL1 by electroporation, and plasmids constructed based on pET-28a were transformed into *E. coli* Transetta (DE3) as the manufacturer’s protocol.

### 2.3 Computational design and analysis of mutation sites

Sequences of the homoserine dehydrogenase from different sources were collected from the UniRef90 database in Universal Protein Resource (UniProt) (https:// www.uniprot.org/,UniProt) (Supplementary Date S1). The alignment function of MEGA software performed multiple sequence alignment, and WebLogo (https://weblogo.berkeley.edu/) was used to analyze the conservatism of amino acids. The homotetramer structure of *Cg*HSD was automatically modeled by using the SWISS-MODEL program (https://swissmodel.expasy.org/) based on the reported crystal structure of homoserine dehydrogenase from *Thiobacillus denitrificans* (PDB code: 3MTJ, 37% identity) (Chen et al., 2015). The enzyme-effector complex was acquired by the flexible docking function of Discovery Studio 2019 software (Accelrys, United States). The amino acid residues on the interaction interface were calculated using PDBePISA (https://www.ebi.ac.uk/pdbe/pisa) (Santos and Passos, 2021). Molecular visualization and figure preparation were performed using PyMOL software (version 1.7.2, Delano Scientific, United States) (Liu et al., 2020). The ΔΔG ^Thr^_binding_ values were calculated by Discovery Studio 2018 (Accelrys, San Diego, United States), similar to the previous study (Liu et al., 2021).

### 2.4 Construction and screening of library

To construct the single-site saturation library, primers with NNN degenerate oligonucleotides were used to randomize the codon of the target site. PCR products covering the randomized sequences and the backbone of the plasmid pEC-*homthrB* were ligated via recombination. These constructed plasmids were transformed into *E. coli* Trans1-T1, respectively. There were more than 300 colonies on each LB solid medium, with 5-fold coverage of the library diversity. All the transformants were collected and used for plasmid extraction. The plasmid library was transformed into LCgL1 via electroporation, and cells were plated onto MM agar plates with 3 g/L of AHV for screening.

### 2.5 Animo acid production by cultivation in 24-deep-well plates

The seed medium of *Cg*HSD and its mutants was TSB. The fermentation medium (pH 7.2) contained 80 g/L glucose, 1 g/L soya peptone, 1 g/L yeast extract, 8 g/L urea, 1 g/L NaCl, 1 g/L KH_2_PO_4_·3H_2_O, 0.45 g/L MgSO_4_·7H_2_O, 0.05 g/L FeSO_4_·7H_2_O, 1 g/L (NH_4_)_2_SO_4_, 0.4 mg/L biotin, 0.1 mg/L vitamin B1, 40 g/L MOPS (pH 7.2). Strains were precultured in 24-deep-well plates with 800 μL TSB medium in each well at 30°C, 800 rpm, and 90% moisture for 8 h. The seed culture was then used to inoculate 24-deep-well plates containing 800 μL TSB medium with 0.5 mM IPTG in each well to an initial OD_600nm_ of 0.06. The plates were cultivated at 30°C, 800 rpm, and 90% moisture for 18 h.

### 2.6 Protein expression and purification

To obtain pure enzyme of *Cg*HSD and its mutants, the strains were cultured with an initial OD_600nm_ of 0.1 in LB at 37°C until OD_600nm_ reached 0.8. Then, IPTG was added to a final concentration of 0.5 mM and induced at 16°C for 16 h. The cells were harvested, washed, and suspended in lysis buffer (100 mM sodium phosphate, 500 mM NaCl, and 20 mM imidazole, pH 7.4). Cells were broken by grinder JXCL-3K (Jingxin, China). The supernatant was collected by centrifuge at 10,000×*g* for 10 min at 4°C. The wild type and the mutants were expressed with a His6-tag at the N terminus. According to the manufacturer’s instructions, the filtered supernatant was loaded on His SpinTrap Kit (Cytiva, United States) for enzyme purification. The elution buffer (pH 7.4) contains 100 mM sodium phosphate and 500 mM imidazole. The purity of the collected protein was checked by SDS-PAGE, and the enzyme concentration was determined with a BCA protein assay kit (Thermo Fisher Scientific, United States).

## 3 Results

### 3.1 Selecting the key residues for semi-rational engineering of *Cg*HSD

**FIGURE 1:**
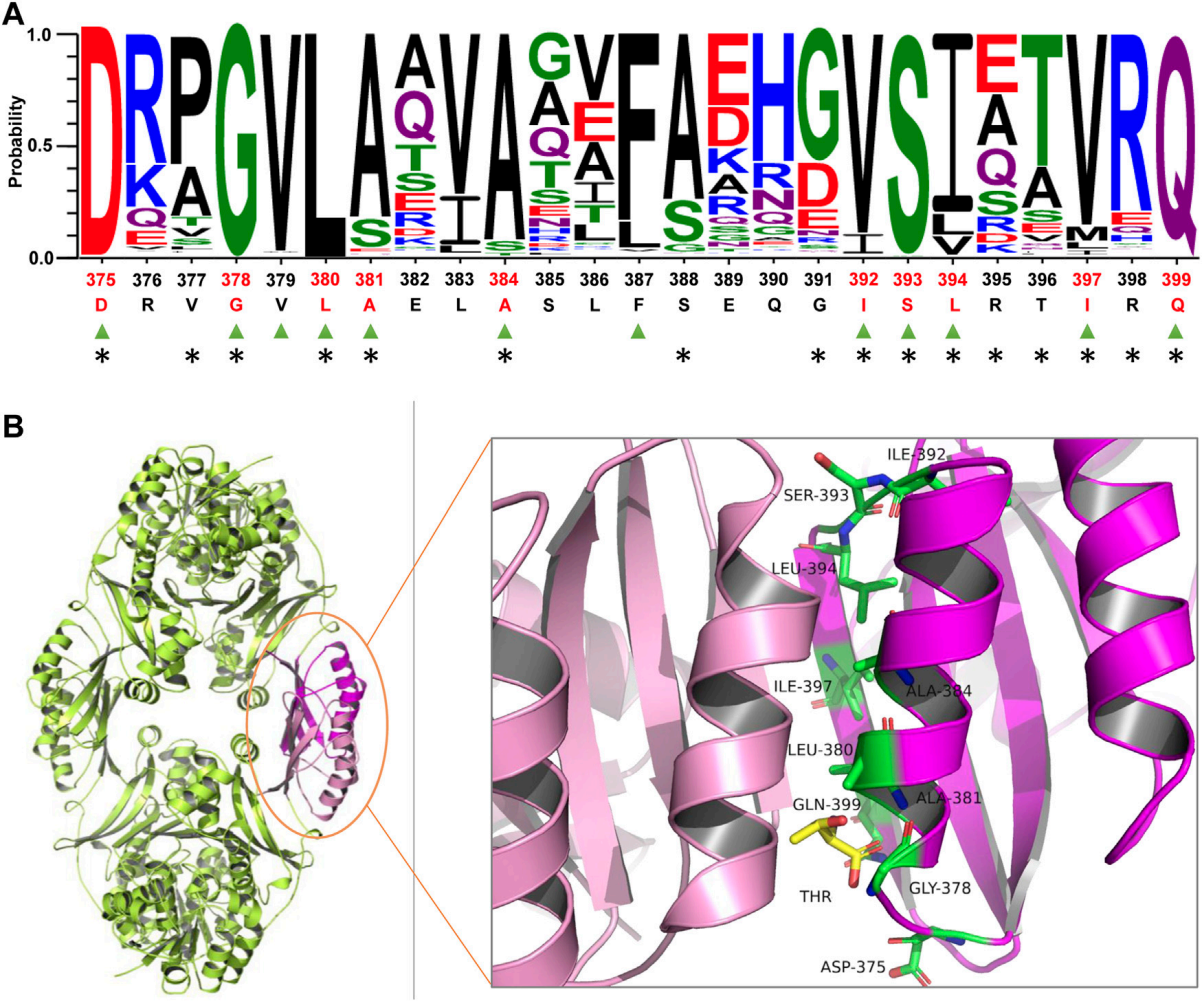
Analyzing conserved amino acid residues at the tetramer interface of homoserine dehydrogenase from *C. glutamicum* (*Cg*HSD). **(A)** Sequence analysis of the central region for allosteric regulatory (375-399 part). The residues labeled by triangles in green are conserved (probability>0.8). The residues located at the tetramer interface are marked with asterisks. The ten conserved residues at the tetramer interface are highlighted in red, including D375, D378, L380, A381, A384, I392, S393, L394, I397 and Q399. The sequences of HSDs used for conservation analysis are provided in Supplementary Date S1. **(B)** The model structure of *Cg*HSD. The model structure was constructed with the crystal structure of HSD from *Thiobacillus denitrificans* (PDB code 3MTJ) as a template (37% sequence identity with *Cg*HSD) using Discovery Studio. The effector l-threonine is indicated in yellow. The ten conserved residues at the tetramer interface are marked in green. The entire structure is shown in Supplementary Figure S3.

Semi-rational enzyme design based on protein structure and sequence information has been proven to significantly improve the efficiency of mutant library construction and screening (Mariz et al., 2021; Victorino da Silva Amatto et al., 2022). The reported HSDs present two oligomeric states, forming a dimer or tetramer. It was presumed that the C-terminal allosteric binding domain of HSD from *Bacillus subtilis* participated in interface interactions responsible for the tetramer formation (Kim et al., 2020). However, the oligomeric state of *Cg*HSD is unknown. We determined the apparent molecular mass with SEC (Supplementary Figure S2); *Cg*HSD seems to be forming a tetramer. Since the crystal structure of *Cg*HSD was unavailable, the homology model of *Cg*HSD was obtained by the software MODELLER with a tetramer HSD from *T*. *denitrificans* (TdHSD, PDB code 3MTJ, 37% sequence identity with *Cg*HSD) as the template (Supplementary Figure S3A). It was predicted that the effector l-threonine binds at the tetramer interface, and the 375–399 residues are the central region for allosteric regulation using flexible docking (Supplementary Figure S3B). The overall structure and effector-protein interaction are similar to a previous report. In their study, several amino acid residues directly interacting with l-threonine were predicted by docking simulation and designed to remove the feedback inhibition. Still, no mutants were suitable for efficient production because of a significant decrease in the specific enzyme activity (Chen et al., 2015). We focused on the conserved amino acids involved in tetramer interface interactions since they are also supposed to play essential roles in maintaining the tetramer structure and feedback inhibition. The sequence conservation and amino acid residues at the tetramer interface were analyzed within the 375–399 region based on 1000 HSD sequences from the UniProt database and the PDBePISA server. Ten conserved amino acid residues (probability>0.8) involved in the tetramer interface interactions were identified, which were D375, D378, L380, A381, A384, I392, S393, L394, I397, and Q399 (Figure 1A and Supplementary Data S1). These residues could be categorized into three groups according to the secondary structure analysis: in detail, D375, D378, I392, S393, and L394 are located at two loop structures; A381 and A384 find at the helix 379–389; I397 and Q399 locate at β-sheet structures (Figure 1B). Since several substitutions at D378 (such as D378E and D378S) have been found to release *Cg*HSD from the feedback inhibition (Sahm et al., 1996; Dong et al., 2016), nine other residues were selected for site-saturation mutagenesis.

### 3.2 Establishing a HTS method for feedback inhibition engineering of *Cg*HSD

**FIGURE 2:**
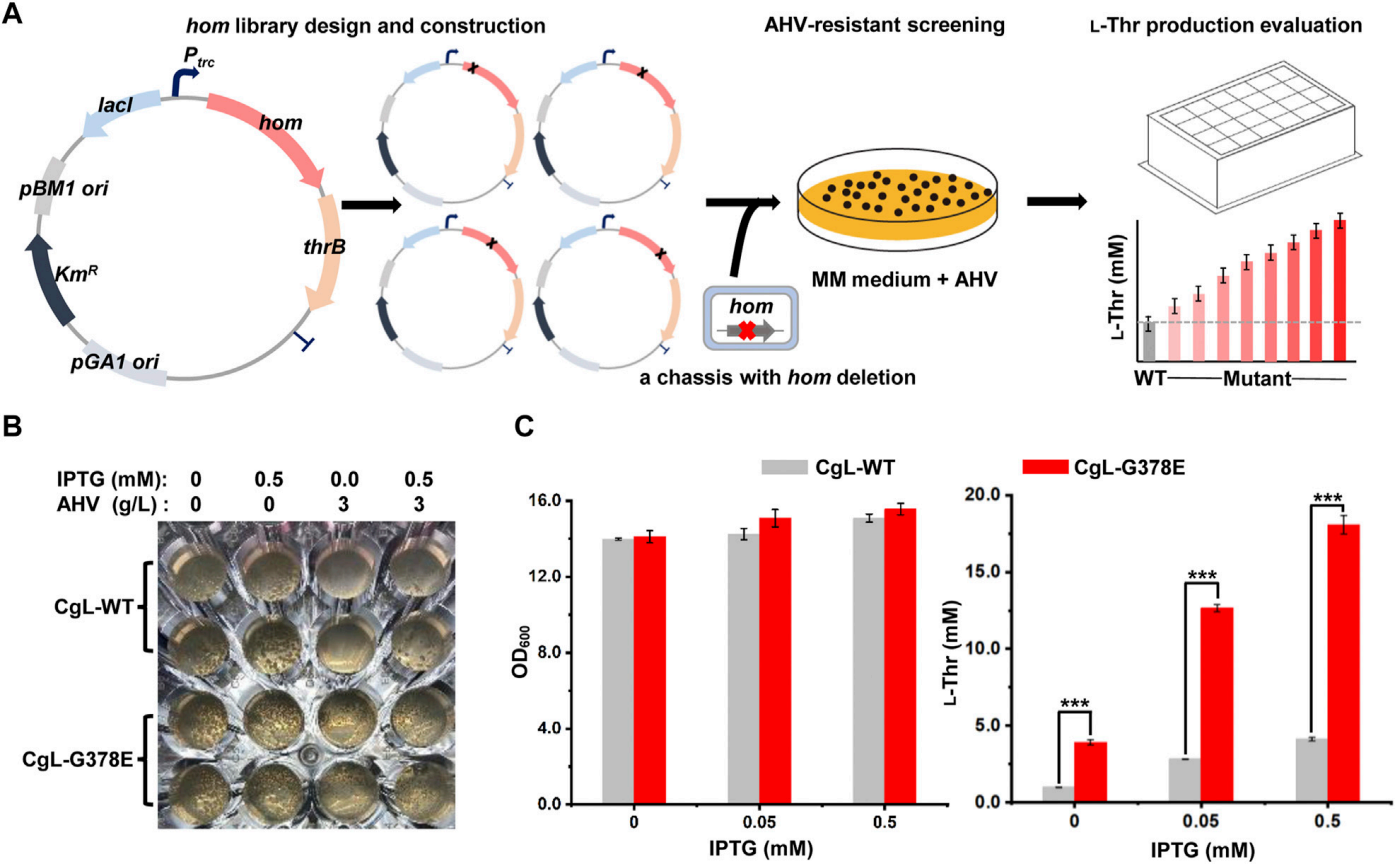
Establishment of a high-throughput screening (HTS) method for screening feedback-resistant mutants of *Cg*HSD. **(A)** Workflow of screening feedback-resistant mutants. The *hom-thrB* operon was expressed on a plasmid, and mutations were introduced to the *hom* gene for mutant library construction. The plasmid mutant libraries were transformed into a chassis strain LCgL1 without the *hom* gene on the chromosome. Then, the resistant variants were screened on minimal media containing the appropriate amount of AHV and IPTG. The resistant mutants were evaluated in 24-deep-well plates. **(B)** Testing the HTS strategy by using the reported G378E mutant. The test experiment was performed on a transparent 48-well plate. The MM solid medium was supplemented with or without IPTG and AHV. The wild type and G378E mutant were inoculated into each well at the same biomass, and the plate was cultivated aerobically at 30°C for 72 h. **(C)** Evaluation of the l-threonine production with different concentrations of IPTG. The 24-deep-well plate fermentation was performed at 30°C for 18 h. Data are presented as mean values +/− SD (n = 3 independent experiments). The *** symbol represents *p* < 0.001, Student’s two-tailed *t*-test.

To efficiently obtain potential candidates from the mutant libraries, a HTS method based on α-Amino-β-hydroxy valeric acid (AHV), an analog of l-threonine (Shiio, 1990), was developed (Figure 2A). Firstly, the *hom-thrB* operon was expressed on a plasmid driven by IPTG-induced promoter *P*_*trc*_, and mutations were introduced to the *hom* gene by the mutant library construction. Secondly, the mutant plasmid libraries were transformed into a chassis strain LCgL1, wherein the *hom* gene on the chromosome was knocked out to avoid heterozygous polymerization between the wild type and the mutant monomer. AHV specifically inhibits the enzyme activity of *Cg*HSD and is incorporated into proteins by biosynthesis, resulting in cell growth inhibition (Shiio, 1990; Rodgers and Shiozawa, 2008). The mutants with feedback-resistant *Cg*HSD can produce more l-threonine and relieve the effects of AHV on cell growth. Therefore, we screened resistant variants on the minimal media containing appropriate AHV and IPTG. Finally, the resistant variants were evaluated for l-threonine production in 24-deep-well plates, and the superior mutants were chosen to verify the mutations at the *hom* gene by DNA sequencing.

To demonstrate the HTS strategy, the expression plasmids of the wild-type *hom* gene and the reported G378E mutant were first used to generate strains CgL-WT and CgL-G378E, respectively. They were then spread and cultivated on the solid medium supplemented with or without IPTG and AHV in a 48-well plate. Both strains grew many colonies (∼60) on plates without AHV. When 3 g/L AHV was added into the medium, the wild-type strain grew significantly fewer colonies than those of the mutant. Interestingly, there was no colony in the wells of the wild-type strain with AHV and without IPTG after 72 h of cultivation, but many colonies were found in the mutant’s wells. The culture condition with 3 g/L AHV and without IPTG is more conducive to screening feedback-resistant mutants (Figure 2B). l-Threonine production was performed in 24-deep-well plates with different IPTG concentrations to evaluate at high yield levels. 0.5 mM IPTG favored the l-threonine production and did not affect the cell growth for both strains in liquid culture. The mutant CgL-G378E produced a significantly higher amount of l-threonine under all conditions than the wide-type strain. It reached the highest l-threonine yield (18.1 mM) with 0.5 mM IPTG, which was 3.4-fold higher than the wide-type strain (4.1 mM) (Figure 2C). These results indicated that the HTS method can successfully distinguish dominant mutants from wild-type *Cg*HSD with feedback inhibition. In fact, we firstly expressed *hom* gene on the plasmid alone to test the HTS strategy. Unfortunately, the wild-type and G378E mutant produced similar l-threonine by cultivation in 24-deep-well plates with different IPTG concentrations (Supplementary Figure S4), exhibiting unsuitability for screening feedback-resistant mutants.

### 3.3 Screening site-saturation mutagenesis libraries and identifying preferred mutants

**FIGURE 3:**
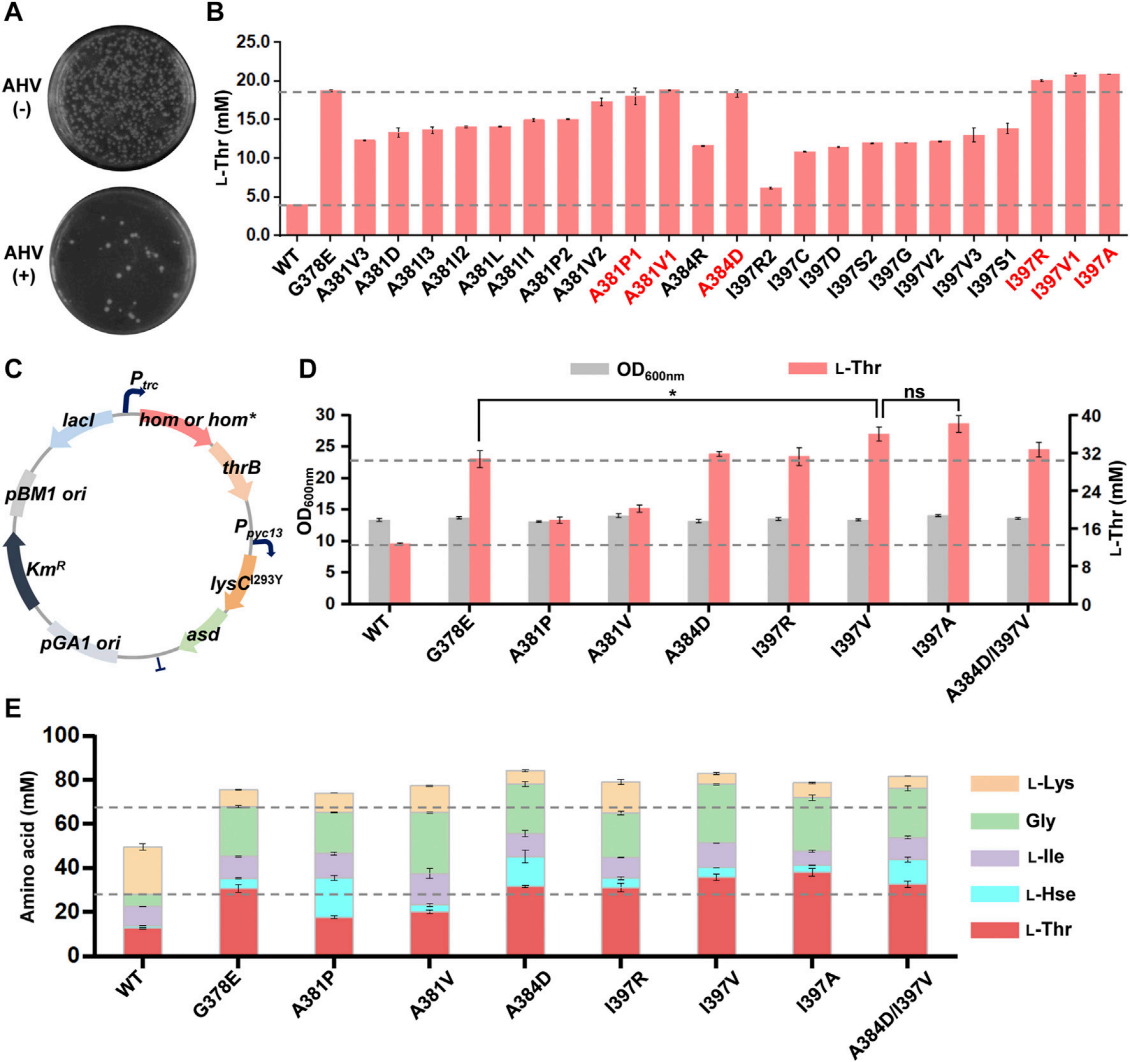
Screening and characterization of feedback-resistant mutants. **(A)** AHV-resistant plate screening for the library of A384 site. The results of A381 and I397 sites are shown in Supplementary Figure S5. The test experiment was performed on an agar plate. The MM solid medium was supplemented with and without 3 g/L AHV. The mutant library was plated onto MM agar plates with and without 3 g/L AHV, respectively, and the plates were cultivated aerobically at 30°C for 72 h. The resistant variants were screened on an agar plate with 3 g/L AHV. **(B)** l-Threonine production of 23 AHV-resistant variants. The preferred mutants are highlighted in red. Different numbers indicate different codon mutations. **(C)** The plasmid map for further overexpression of *lysC* ^I293Y^-*asd* operon. **(D)** Effect of different mutations on the growth and l-threonine production after further overexpression of *lysC* ^I293Y^-*asd* operon. **(E)** Effect of different mutations on the production of other related aspartate family amino acids after further overexpression of the *lysC* ^I293Y^-*asd* operon. The 24-deep-well plate fermentation was performed at 30°C for 18 h with 0.5 mM IPTG. l-Thr, l-threonine; l-Hse, l-homoserine; l-Ile, l-isoleucine; Gly, glycine; l-Lys, l-lysine. Data are presented as mean values +/− SD (*n* = 3 independent experiments). The * symbol represents *p* < 0.05, ns represents *p* > 0.05, Student’s two-tailed *t*-test.

We used primers with NNN-degenerate codons to introduce saturated mutations at the selected nine residues of the *C. glutamicum hom* gene and generated nine plasmid libraries in *E. coli*, respectively, of each library comprising >300 transformants and covering >95% probability. The mutant plasmid libraries were then transformed into the chassis *C. glutamicum* strain LCgL1, respectively, and more than 300 transformants were used for screening resistant variants on agar plates with the medium mentioned above and culture condition. Three mutant libraries (A381, A384, and I397) grew some colonies after 72 h of cultivation (A381 in Figure 3A; A384 and I397 in Supplementary Figure S5). DNA sequencing revealed 23 AHV-resistant variants with different codons, including 13 amino acid substitutions (the detail substitutions were shown in Supplementary Table S4). All the variants were evaluated for l-threonine fermentation in liquid culture with 0.5 mM IPTG in 24-deep-well plates, and the strain harboring the wild-type *hom* gene was used as a control. Compared to the wild type, all mutant variants produced 0.6- to 4.3-fold higher l-threonine after fermentation for 18 h (Figure 3B), which suggested the HTS method established in this study can efficiently select superior l-threonine-producing mutants with possible feedback-resistant properties. Compared with the positive control G378E, three substitutions (I397R, I397V, and I397A, *p* < 0.05) produced a slightly higher amount of l-threonine, and three substitutions (A381P, A381V1, and A384D, *p* > 0.05) gave rise to an equivalent amount.

To further explore the potential benefit of six dominant substitutions for l-threonine production, another chassis strain with a higher l-threonine production level was constructed as follows. The *lysC* ^I293Y^ *-asd* operon for enhancing precursor supply was further overexpressed under the control of a strong constitutive promoter *P*_*pyc*_-13 (Liu et al., 2022) on the plasmid with the *hom-thrB* operon, and then six substitutions were introduced, respectively (Figure 3C). Compared to the wild type, all six substitutions increased l-threonine production in the new chassis under the same testing conditions (Figure 3D). Moreover, I397V and I397A substitutions led to an appropriate 20% higher l-threonine (*p* < 0.05) than the positive control G378E substitution and equivalent amounts for A384D and I397R substitutions. Several synthetic metabolism-related amino acids were also determined to investigate further the effect of different mutants on other amino acid production (Figure 3E). These amino acids can be converted into equimolar amounts. In comparison to the wild-type *Cg*HSD, all mutants significantly increased the output of *Cg*HSD-dependent amino acids (l-threonine, l-homoserine, l-isoleucine, and glycine) along with higher total yield, suggesting the metabolic flow was shifted to the l-homoserine direction. Compared to the positive control G378E, both A384D and I397V substitutions also generated more *Cg*HSD-dependent amino acids, with amounts of ∼78 mM (increased by ∼15% of G378E at 68 mM). The double mutations (A384D and I397V) did not increase amino acid production further. The results suggested the advantage of A384D and I397V substitutions compared with the ever-reported best mutant G378E.

### 3.4 *In vitro* enzyme characterization of mutant *Cg*HSDs

**FIGURE 4:**
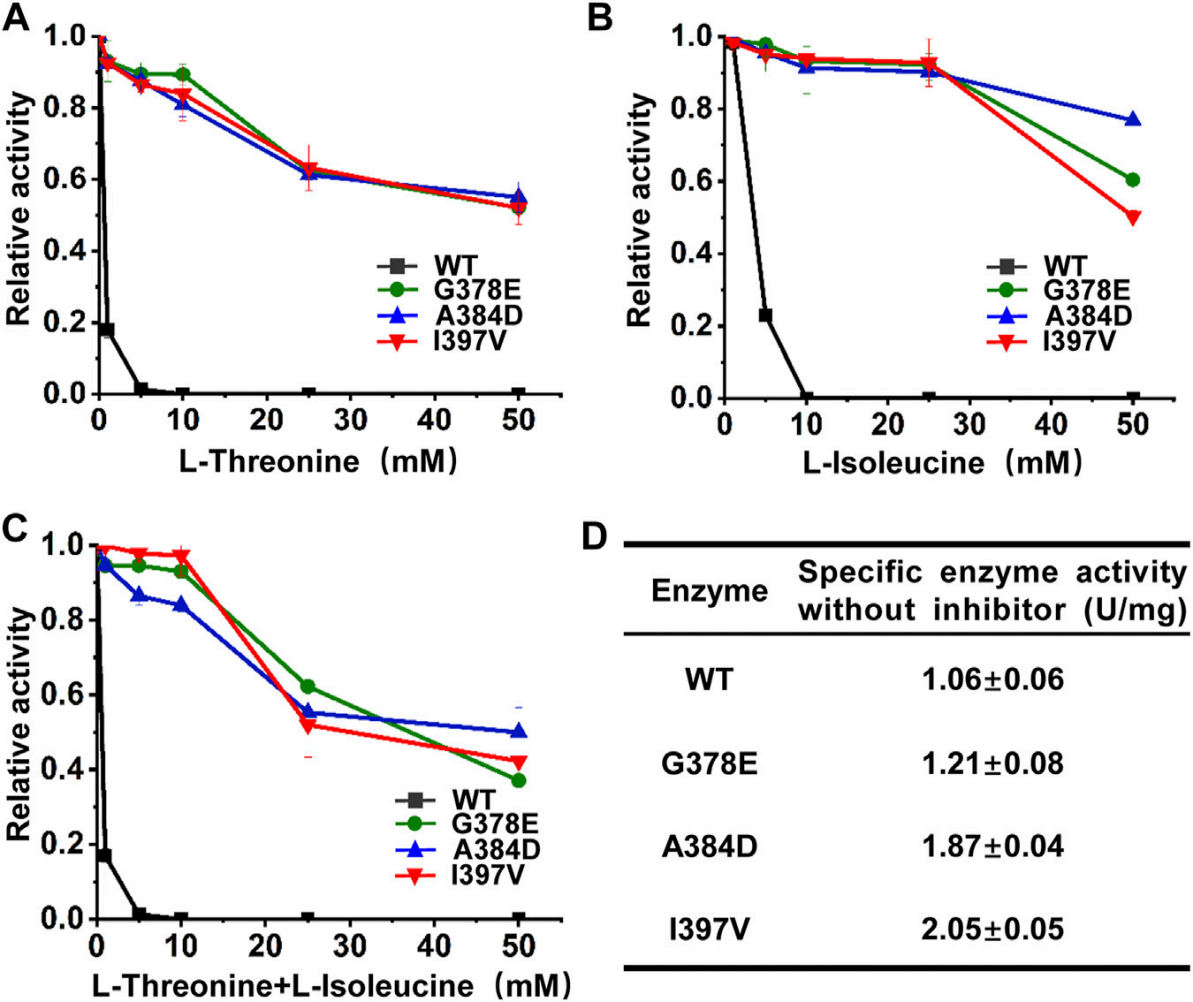
Inhibition profiles of purified *Cg*HSD and its mutants. **(A)** Inhibition profiles of *Cg*HSD and its mutants by l-threonine. **(B)** Inhibition profiles of *Cg*HSD and its mutants by l-isoleucine. **(C)** Inhibition profiles of *Cg*HSD and its mutants by l-threonine and l-isoleucine. Two inhibitors were added simultaneously with the same concentration (0–50 mM). **(D)** The specific enzyme activities of the *Cg*HSD and its mutants without inhibitors. The inhibition curves are expressed as relative activities, taking the activity of each enzyme without inhibitors as 100%. One unit of enzyme activity was defined as the amount of enzyme needed that produced 1 μmol NADPH per minute. Detailed assay conditions are described in the methods section. Data are presented as mean values +/− SD (*n* = 3 independent experiments).

The wild-type and mutant *Cg*HSDs were overexpressed in *E. coli* with His-tag at the N terminus and purified by Ni–NTA affinity chromatography. All *Cg*HSDs showed well-expressed soluble proteins and high-purity enzymes were obtained successfully (Supplementary Figure S6). The enzyme activities and the inhibition profiles of the enzymes were determined. Regarding l-threonine inhibition (Figure 4A), the wild-type *Cg*HSD was dramatically inhibited by 1 mM l-threonine, and the activity loss was more than 80%. Two screened mutant *Cg*HSDs (I397V and A384D) significantly reduced the inhibitory effect of l-threonine, which performed as well as the positive control G378E. The mutant *Cg*HSDs with A384D and I397V substitutions kept more than 90% of their activity at 10 mM l-threonine and more than 50% of their activity even at 50 mM l-threonine, respectively, suggesting that A384 and I397 are essential sites for l-threonine binding. Speaking of the isoleucine inhibition (Figure 4B), the wild-type *Cg*HSD was also strongly inhibited by 5 mM l-isoleucine, and the activity loss was more than 75%. The mutants (A384D and I397V, respectively) retained more than 90% of their activity at 25 mM l-isoleucine, which behaved as well as the positive control G378E. Moreover, the A384D successfully maintained 76.5% activity, which surpassed the G378E (61.0%) and I397V (50.1%) mutants at 50 mM l-isoleucine. The simultaneous addition of l-threonine and l-isoleucine did not result in a sharp decline compared to single inhibition (Figure 4C). Notably, the retained activity of the A384D mutant was 50.7% when 50 mM l-threonine and 50 mM l-isoleucine were added simultaneously. The specific enzyme activities of the purified enzymes were measured in the absence of inhibitors. The substitutions A384D and I397V significantly transcended the positive control G378E and gave rise to 54.0% and 69.2% higher specific activities, respectively (Figure 4D). These results suggested that introducing A384D or I397V substitutions into *Cg*HSD efficiently relieves the feedback inhibition and increases the specific enzyme activity.

Because the mutations were located at the tetramer interface, we also carried out SEC to determine the oligomeric states of the wild-type and mutant *Cg*HSDs. The peaks of all *Cg*HSDs emerged at 11.8 mL of elution volume, which was 4.5 times greater than the actual molecular mass of monomeric *Cg*HSD (46.4 kDa) from the standard curve (Supplementary Figure S2). These results showed that all *Cg*HSDs exist as a tetramer in solution. We suggested that keeping the tetramer structure is critical for the increases in specific enzyme activities.

## 4 Discussion

Protein engineering has been widely used to relieve feedback inhibition of key enzymes in amino acid biosynthesis pathways (Li et al., 2017; Shimizu and Matsuoka, 2022). Redesigning the original non-covalent bond network around the effector is an effective method to adjust the binding efficiency of the effector (Wang et al., 2018). However, as a multimer protein, *Cg*HSD, the key enzyme in the synthesis of various l-aspartate family amino acids and derivatives in *C. glutamicum*, it is difficult to obtain feedback inhibition-resistant variants without significant loss in enzyme activity by the rational protein design (Chen et al., 2015). To date, only the G378E mutant of *Cg*HSD (retained activity of 52.7% at 50 mM l-threonine, Figure 4A) was generated by traditional strain mutagenesis (Sahm et al., 1996), which has been widely employed to develop the producers of downstream products, such as l-threonine and l-isoleucine (Vogt et al., 2015; Wei et al., 2018). Metabolic engineering was also made to enhance the catalytic reaction by *Cg*HSD for l-homoserine production in *C. glutamicum* by overexpression of a bifunctional analog enzyme from *E. coli* (Li et al., 2023). In the present study, we demonstrated a promising strategy to engineer the allosteric inhibition in amino acid biosynthesis by direct coupling feedback inhibition with the analog resistance and establishing a subsequent HTS method to screen the semi-rational designed libraries efficiently. We obtained two novel mutants (A384D and I397V) compared to the known mutant (G378E, both mutants successfully relieved the feedback inhibition of l-threonine and l-isoleucine and increased specific enzyme activities without inhibitors (Figure 4), which was demonstrated to produce higher HSD-dependent amino acids in the same evaluation background (Figure 3E). We provide good *Cg*HSD candidates for the production improvement of these amino acids in *C. glutamicum*.

We also offer positive mutants with efficient feedback inhibition resistance and high enzyme activities, which may help to elucidate the structure-function relationship of *Cg*HSD. We calculated the changes in l-threonine binding free energy (ΔΔG^Thr^_binding_) by computational analysis, and all variants displayed unbeneficial ΔΔG^Thr^_binding_ values (>2.5 kJ/mol) on l-threonine binding (Supplementary Table S5) as expected. Considering the l-threonine binding pocket was located at the tetramer interface of two subunits and no direct non-covalent bond interactions were found between the mutant sites and l-threonine, we believed that the A384D and I397V substitutions may alter the original binding mode of neighboring subunits, and indirectly reorganize the shape of the binding pocket, thus impair the l-threonine recognition and combination. The changes in non-covalent interaction by introducing dominant substitutions (A384D and I397V, respectively) were also predicted. The original residues A384 and I397 were distributed in the interface of subunits A and D, A384D substitution increased an additional charge-charge repulsion interaction (Supplementary Figure S7A, B), and I397V substitution reduced a conventional hydrogen bond formed between 397 and T396 (Supplementary Figure S7C, D). These analyses of local structural perturbations further support our hypotheses. In particular, these substitutions did not disrupt the native tetramer structure (Supplementary Figure S2), which could partially explain why the mutants increased the specific enzyme activities without inhibitors. To give a more accurate understanding, crystallographic analysis of *Cg*HSD and millisecond scale molecular simulations could be carried out in the future.

In conclusion, we developed an efficient approach to engineering the feedback inhibition of *Cg*HSD by combining a semi-rational design and a HTS method. The obtained mutants displayed excellent feedback inhibition resistance and high specific enzyme activity, providing high-performance catalytic elements for the efficient biosynthesis of various amino acids with l-aspartate 4-semialdehyde as a common precursor. The design and screening strategies can also inspire the engineering of other allosteric regulatory enzymes in amino acid biosynthesis.

## Data Availability

The original contributions presented in the study are included in the article/Supplementary Material, further inquiries can be directed to the corresponding authors.

## References

[B1] Akai S. Ikushiro H. Sawai T. Yano T. Kamiya N. Miyahara I. (2019). The crystal structure of homoserine dehydrogenase complexed with l-homoserine and NADPH in a closed form. J. Biochem. 165 (2), 185–195. 10.1093/jb/mvy094 30423116

[B2] Chen Z. Meyer W. Rappert S. Sun J. Zeng A. P. (2011a). Coevolutionary analysis enabled rational deregulation of allosteric enzyme inhibition in *Corynebacterium glutamicum* for lysine production. Appl. Environ. Microbiol. 77 (13), 4352–4360. 10.1128/aem.02912-10 21531824 PMC3127711

[B3] Chen Z. Rappert S. Sun J. Zeng A. P. (2011b). Integrating molecular dynamics and co-evolutionary analysis for reliable target prediction and deregulation of the allosteric inhibition of aspartokinase for amino acid production. J. Biotechnol. 154 (4), 248–254. 10.1016/j.jbiotec.2011.05.005 21609739

[B4] Chen Z. Rappert S. Zeng A. P. (2015). Rational design of allosteric regulation of homoserine dehydrogenase by a nonnatural inhibitor l-lysine. ACS Synth. Biol. 4 (2), 126–131. 10.1021/sb400133g 24344690

[B5] Dong X. Zhao Y. Zhao J. Wang X. (2016). Characterization of aspartate kinase and homoserine dehydrogenase from *Corynebacterium glutamicum* IWJ001 and systematic investigation of l-isoleucine biosynthesis. J. Ind. Microbiol. Biotechnol. 43 (6), 873–885. 10.1007/s10295-016-1763-5 27033538

[B6] Geng F. Chen Z. Zheng P. Sun J. Zeng A. P. (2013). Exploring the allosteric mechanism of dihydrodipicolinate synthase by reverse engineering of the allosteric inhibitor binding sites and its application for lysine production. Appl. Microbiol. Biotechnol. 97 (5), 1963–1971. 10.1007/s00253-012-4062-8 22644522

[B7] Kim D. H. Nguyen Q. T. Ko G. S. Yang J. K. (2020). Molecular and enzymatic features of homoserine dehydrogenase from *Bacillus subtilis* . J. Microbiol. Biotechnol. 30 (12), 1905–1911. 10.4014/jmb.2004.04060 33046675 PMC9728202

[B8] Leander M. Yuan Y. Meger A. Cui Q. Raman S. (2020). Functional plasticity and evolutionary adaptation of allosteric regulation. Proc. Natl. Acad. Sci. U. S. A. 117 (41), 25445–25454. 10.1073/pnas.2002613117 32999067 PMC7568325

[B9] Li N. Li L. Yu S. Zhou J. (2023). Dual-channel glycolysis balances cofactor supply for l-homoserine biosynthesis in *Corynebacterium glutamicum* . Bioresour. Technol. 369, 128473. 10.1016/j.biortech.2022.128473 36509305

[B10] Li Y. Wei H. Wang T. Xu Q. Zhang C. Fan X. (2017). Current status on metabolic engineering for the production of l-aspartate family amino acids and derivatives. Bioresour. Technol. 245 (Pt B), 1588–1602. 10.1016/j.biortech.2017.05.145 28579173

[B11] Liu J. Liu M. Shi T. Sun G. Gao N. Zhao X. (2022). CRISPR-assisted rational flux-tuning and arrayed CRISPRi screening of an l-proline exporter for l-proline hyperproduction. Nat. Commun. 13 (1), 891. 10.1038/s41467-022-28501-7 35173152 PMC8850433

[B12] Liu J. Wang Y. Lu Y. Zheng P. Sun J. Ma Y. (2017). Development of a CRISPR/Cas9 genome editing toolbox for *Corynebacterium glutamicum* . Microb. Cell Fact. 16 (1), 205. 10.1186/s12934-017-0815-5 29145843 PMC5693361

[B13] Liu Z. Fu X. Yuan M. Liang Q. Zhu C. Mou H. (2021). Surface charged amino acid-based strategy for rational engineering of kinetic stability and specific activity of enzymes: linking experiments with computational modeling. Int. J. Biol. Macromol. 182, 228–236. 10.1016/j.ijbiomac.2021.03.198 33831449

[B14] Liu Z. Ning C. Yuan M. Yang S. Wei X. Xiao M. (2020). High-level expression of a thermophilic and acidophilic β-mannanase from Aspergillus kawachii IFO 4308 with significant potential in mannooligosaccharide preparation. Bioresour. Technol. 295, 122257. 10.1016/j.biortech.2019.122257 31648129

[B15] Mariz B. P. Carvalho S. Batalha I. L. Pina A. S. (2021). Artificial enzymes bringing together computational design and directed evolution. Org. Biomol. Chem. 19 (9), 1915–1925. 10.1039/d0ob02143a 33443278

[B16] Navratna V. Reddy G. Gopal B. (2015). Structural basis for the catalytic mechanism of homoserine dehydrogenase. Acta Crystallogr. Sect. D. Biol. Crystallogr. 71 (Pt 5), 1216–1225. 10.1107/s1399004715004617 25945586

[B17] Rodgers K. J. Shiozawa N. (2008). Misincorporation of amino acid analogues into proteins by biosynthesis. Int. J. Biochem. Cell Biol. 40 (8), 1452–1466. 10.1016/j.biocel.2008.01.009 18329946

[B18] Ruan Y. Zhu L. Li Q. (2015). Improving the electro-transformation efficiency of Corynebacterium glutamicum by weakening its cell wall and increasing the cytoplasmic membrane fluidity. Biotechnol. Lett. 37 (12), 2445–2452. 10.1007/s10529-015-1934-x 26354854

[B19] Sahm H. Eggeling L. Eikmanns B. Krämer R. (1996). Construction of l-lysine-l-threonine-and l-isoleucine-overproducing strains of *Corynebacterium glutamicum* . Ann. N. Y. Acad. Sci. 782, 25–39. 10.1111/j.1749-6632.1996.tb40544.x 8659901

[B20] Sander T. Farke N. Diehl C. Kuntz M. Glatter T. Link H. (2019). Allosteric feedback inhibition enables robust amino acid biosynthesis in *E. coli* by enforcing enzyme overabundance. Cell Syst. 8 (1), 66–75.e8. 10.1016/j.cels.2018.12.005 30638812 PMC6345581

[B21] Santos J. C. Passos G. A. (2021). The high infectivity of SARS-CoV-2 B.1.1.7 is associated with increased interaction force between Spike-ACE2 caused by the viral N501Y mutation. 10.1101/2020.12.29.424708

[B22] Shiio I. (1990). Threonine production by dihydrodipicolinate synthase-defective mutants of *Brevibacterium flavum* . Biotechnol. Adv. 8 (1), 97–103. 10.1016/0734-9750(90)90006-w 14545904

[B23] Shimizu K. Matsuoka Y. (2022). Feedback regulation and coordination of the main metabolism for bacterial growth and metabolic engineering for amino acid fermentation. Biotechnol. Adv. 55, 107887. 10.1016/j.biotechadv.2021.107887 34921951

[B24] Victorino da Silva Amatto I. Gonsales da Rosa-Garzon N. Antônio de Oliveira Simões F. Santiago F. Pereira da Silva Leite N. Raspante Martins J. (2022). Enzyme engineering and its industrial applications. Biotechnol. Appl. Biochem. 69 (2), 389–409. 10.1002/bab.2117 33555054

[B25] Vogt M. Krumbach K. Bang W. G. van Ooyen J. Noack S. Klein B. (2015). The contest for precursors: channelling l-isoleucine synthesis in *Corynebacterium glutamicum* without byproduct formation. Appl. Microbiol. Biotechnol. 99 (2), 791–800. 10.1007/s00253-014-6109-5 25301583

[B26] Wang Y. Jiang Y. Ding S. Li J. Song N. Ren Y. (2018). Small molecule inhibitors reveal allosteric regulation of USP14 via steric blockade. Cell Res. 28 (12), 1186–1194. 10.1038/s41422-018-0091-x 30254335 PMC6274642

[B27] Wei L. Xu N. Wang Y. Zhou W. Han G. Ma Y. (2018). Promoter library-based module combination (PLMC) technology for optimization of threonine biosynthesis in *Corynebacterium glutamicum* . Appl. Microbiol. Biotechnol. 102 (9), 4117–4130. 10.1007/s00253-018-8911-y 29564525

[B28] Xie S. Shen B. Zhang C. Huang X. Zhang Y. (2014). sgRNAcas9: a software package for designing CRISPR sgRNA and evaluating potential off-target cleavage sites. PLoS One 9 (6), e100448. 10.1371/journal.pone.0100448 24956386 PMC4067335

