# Supplementary material for: Engineering allosteric inhibition of homoserine dehydrogenase by semi-rational saturation mutagenesis screening
Source: Front Bioeng Biotechnol. 2024 Jan 3;11:1336215. doi: 10.3389/fbioe.2023.1336215 (PMC10791936; doi:10.3389/fbioe.2023.1336215)
Supplement: Supplementary file 2 [file DataSheet1.docx]

***Supplementary Material***

**Engineering Allosteric Inhibition of Homoserine Dehydrogenase by Semi-rational Saturation Mutagenesis Screening**

**Xinyang Liu^1,2,3,4^*^†^*, Jiao Liu^2,3^*^†^*, Zhemin Liu^2,3^, Qianqian Qiao^1,2,3,4^, Xiaomeng Ni, Jinxing Yang^2,3^, Guannan Sun^2,3^, Fanghe Li^2,3^, Wenjuan Zhou^2,3^, Xuan Guo^2,3^, Jiuzhou Chen^2,3^, Shiru Jia^1,4^, Yu Zheng^1,4^, Ping Zheng^1,2,3*^, Jibin Sun^1,2,3*^**

^1^College of Biotechnology, Tianjin University of Science and Technology, Tianjin 300457, China.

^2^Key Laboratory of Engineering Biology for Low-carbon Manufacturing, Tianjin Institute of Industrial Biotechnology, Chinese Academy of Sciences, Tianjin, 300308, China

^3^National Technology Innovation Center of Synthetic Biology, Tianjin 300308, China.

^4^State Key Laboratory of Food Nutrition and Safety, Tianjin University of Science and Technology, Tianjin 300457, China.

***Corresponding authors**

Dr. Jibin Sun

[sun_jb@tib.cas.cn](mailto:sun_jb@tib.cas.cn)

Dr. Ping Zheng

[zheng_p@tib.cas.cn](mailto:zheng_p@tib.cas.cn)

# Supplementary Materials and Methods

## Enzyme Activity Assays

The activity of *Cg*HSD and its mutants were determined based on a previously described method (Dong et al., 2016), but with minor modifications. The reaction mixture of 200 μL contained 100 mM Tris–HCl (pH 8.0), 60 mM l- homoserine, 1.2 mM NADP^+^, different concentrations of effectors, and an appropriate amount of enzyme. The reaction was started by adding purified enzymes, and the change of NADPH was measured at 340 nm using a SpectraMax ABS Plus (Molecular Devices, USA) at 30°C. One unit of enzyme activity was defined as the amount of enzyme needed that produced one micromole NADPH per minute.

## Analytical Methods

Cell growth of *C. glutamicum* was monitored by measuring OD_600nm_ using the Tecan Infinite® 200 Pro microplate reader (Tecan, China). Analysis of the concentrations of amino acids was carried out by HPLC. The HPLC system comprises a Prominence UFLC (Shimadzu, Japan) equipped with a ZORBAX Eclipse AAA column (4.6 mm × 150 mm, 5 μm, Agilent Technologies, USA) and a UV detector. Eluent and buffer were as described previously(Liu et al., 2022). SEC was carried out using AKTA Purifier 10 equipped with a Superdex 200 increase 10/300 GL column (Cytiva, USA) at 4°C with a flow rate of 0.4 mLmin^-1^. The samples were loaded onto the column pre-equilibrated with 100 mM sodium phosphate (pH 7.4).

# Supplementary Figures and Tables

## Supplementary Figures

**
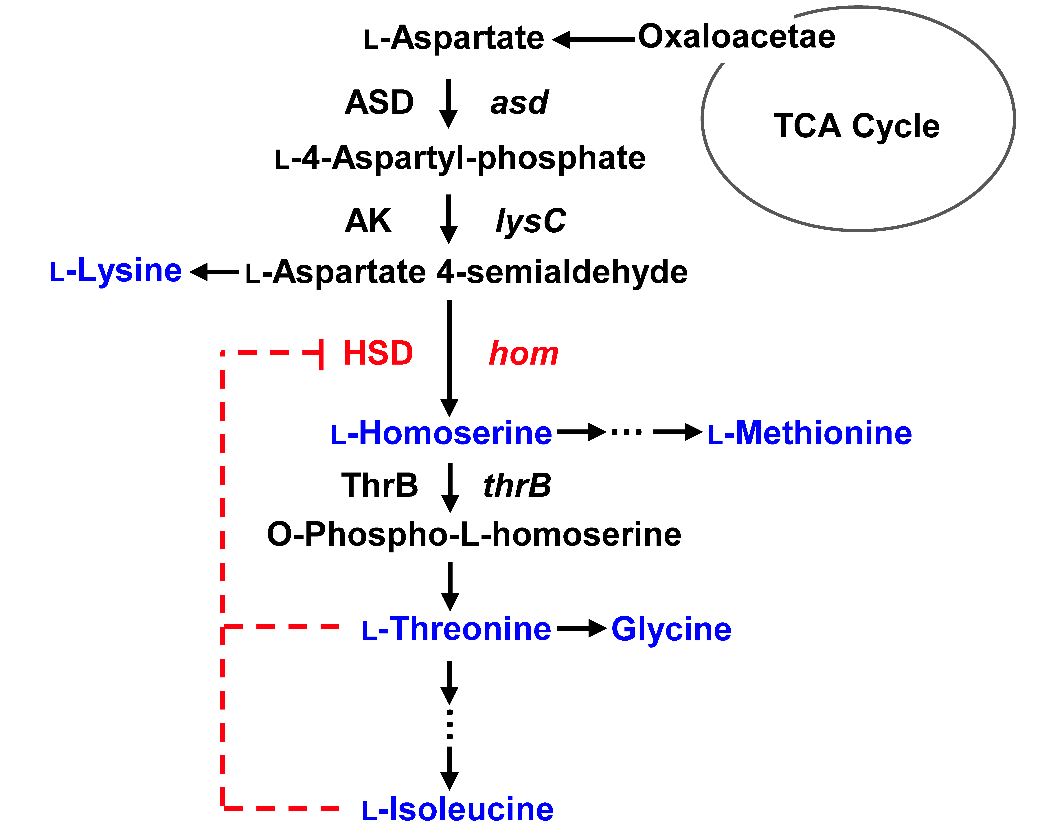
**

**Supplementary Figure 1.** Amino acids and derivatives metabolic synthesis pathways related to *Cg*HSD. The dashed line in red represents the feedback inhibition of the effectors. Amino acids in blue are the competitive or downstream products of the *Cg*HSD.


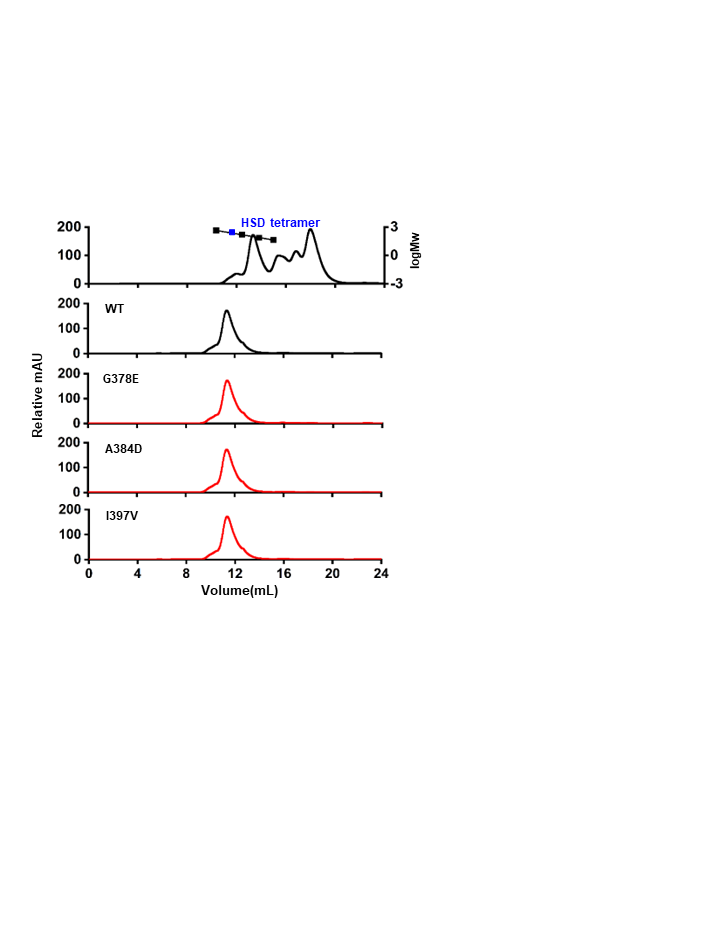


**Supplementary Figure 2.** Size-exclusion chromatography (SEC) analysis. The molecular mass of purified *Cg*HSD and its mutants was measured in size-exclusion column chromatography to investigate the oligomeric state in the solution. The peak corresponds to *Cg*HSD, and its mutants eluted at 11.8 mL.


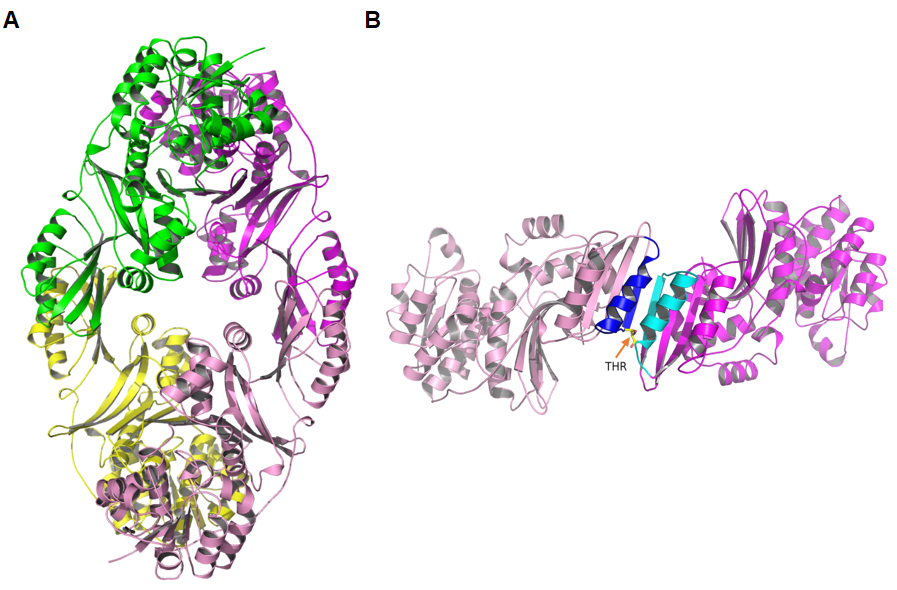


**Supplementary Figure 3.** Homology modeling of *Cg*HSD. **(A)** The predicted tetrameric structure. Different subunits are shown with different colors. **(B)** The l-threonine binding site and the central region for allosteric regulatory (375-399 residues). The 375-399 residues of two neighboring subunits are indicated in blue and cyan, respectively. THR, l-threonine.


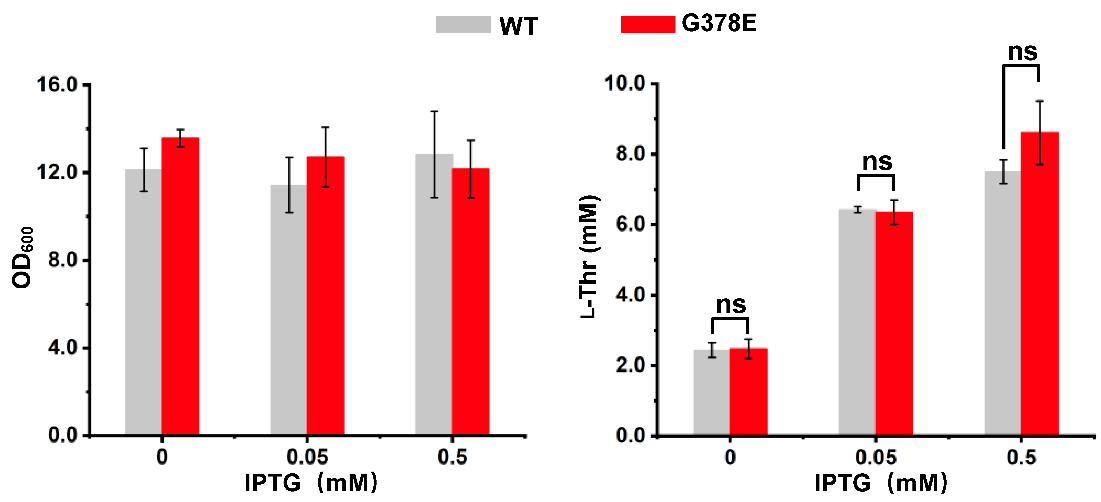


**Supplementary Figure 4.** Evaluating l-threonine production of the wild-type and G378E mutant based the alone overexpression of *hom* gene. The 24-deep-well plate fermentation was performed at 30°C for 18 h with different concentrations of IPTG. Data are presented as mean values +/− SD (n = 3 independent experiments). The ns symbol represents *P* > 0.05, Student’s two-tailed *t*-test.


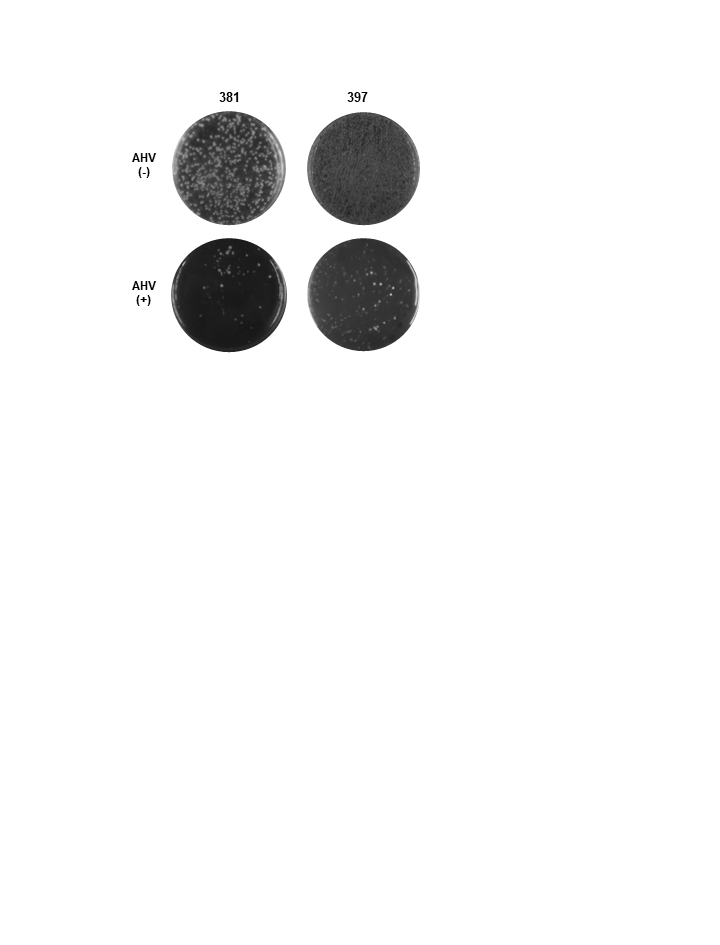


**Supplementary Figure 5.** The screening for the libraries of A381 and I397 sites. The test experiment was performed on an agar plate. The MM solid medium was supplemented with and without 3 g/L AHV. The mutant library was plated onto MM agar plates with and without 3 g/L AHV, respectively, and the plates were cultivated aerobically at 30°C for 72 h. The resistant variants were screened on an agar plate with 3 g/L AHV.


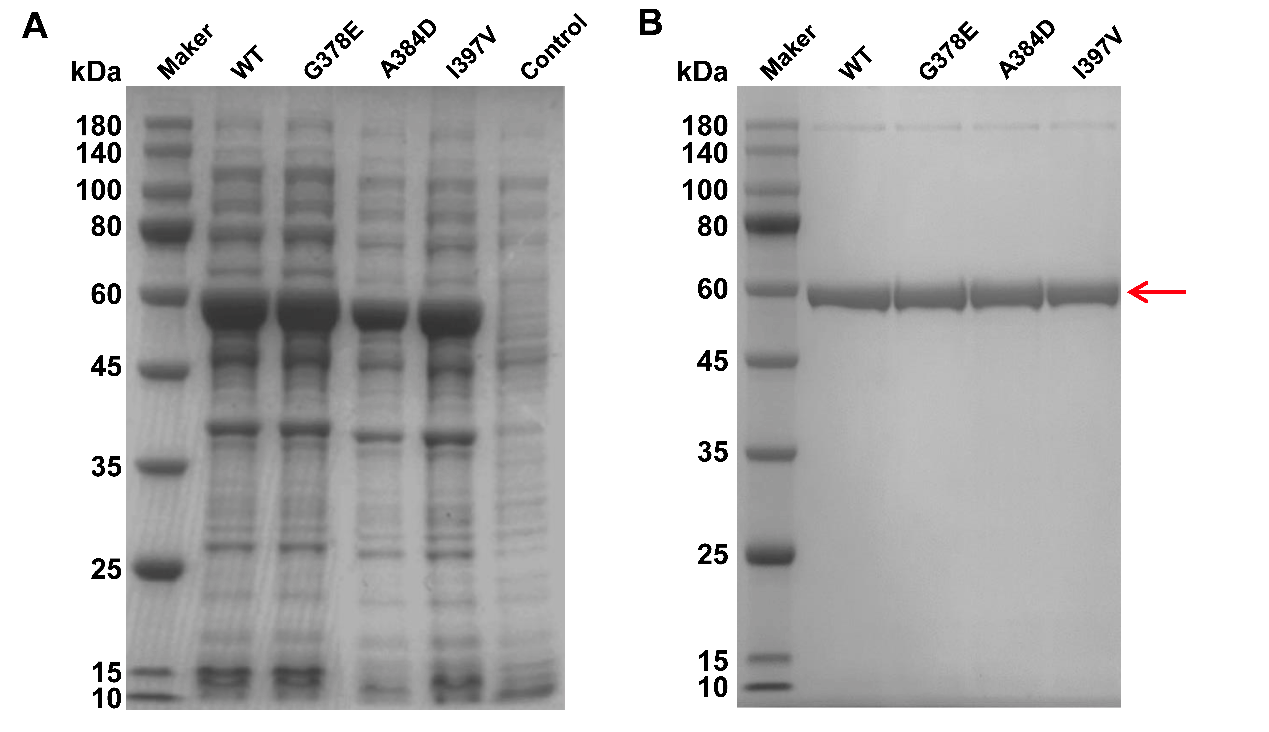


**Supplementary Figure 6.** Soluble expression and purification of *Cg*HSDs. **(A)** SDS-PAGE analysis of *Cg*HSDs soluble expression in *E. coli* Transetta (DE3). Control, *E. coli* Transetta (DE3) with empty vector. **(B)** SDS-PAGE analysis of purified *Cg*HSDs. The purified *Cg*HSDs are denoted by red arrow.


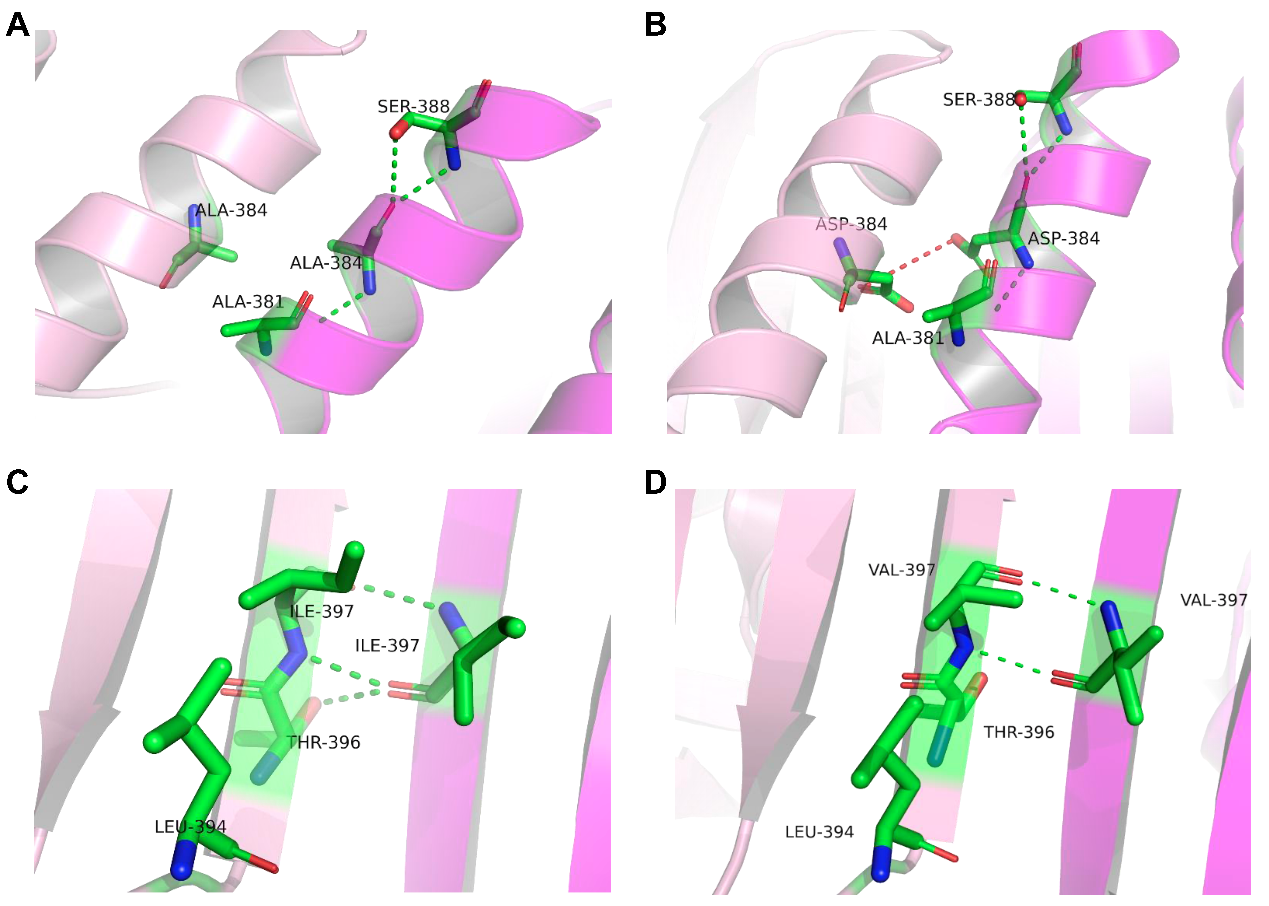


**Supplementary Figure 7.** Non-covalent interactions of two mutation residues (384 and 397) in the wild-type and corresponding dominant mutants. **(A)** A384 in the wild-type. **(B)** D384 in the A384D mutant. **(C)** I397 in the wild-type. **(D)** V397 in the I397V mutant. The green dashed lines indicate conventional hydrogen bonds, and the red ones show charge-charge repulsion forces.

## Supplementary Tables

**Supplementary Table 1.** Strains used in this study

| **Strain** | **Description** | **Source** |
| --- | --- | --- |
| ***E. coli*** |  |  |
| Trans1-T1 | General cloning host | Transgen Biotech |
| Transetta (DE3) | Expression host, derivative carrying chloramphenicol resistant plasmid BL21 | Transgen Biotech |
| ***C. glutamicum*** |  |  |
| ATCC 13032 | Wild-type strain | ATCC |
| ZCgLJ6 | ATCC 13032 derivative with I293Y mutation of *lysC* | Lab stock |
| LCgL1 | ZCgLJ6 derivative with deletion of *hom* | This study |
| CgL-WT | LCgL1 derivative harboring pEC-*homthrB* plasmid | This study |
| CgL-G378E | LCgL1 derivative harboring pEC-*hom*^G378E^*thrB* plasmid | This study |
| CgH-WT | LCgL1 derivative harboring pEC-*HTLA* plasmid | This study |
| CgH-G378E | LCgL1 derivative harboring pEC-*H*^G378E^*TLA* plasmid | This study |
| CgH-A381V | LCgL1 derivative harboring pEC-*H*^A381V^*TLA* plasmid | This study |
| CgH-A381P | LCgL1 derivative harboring pEC-*H*^A381P^*TLA* plasmid | This study |
| CgH-A384D | LCgL1 derivative harboring pEC-*H*^A384D^*TLA* plasmid | This study |
| CgH-I397R | LCgL1 derivative harboring pEC-*H*^I397R^*TLA* plasmid | This study |
| CgH-I397A | LCgL1 derivative harboring pEC-*H*^I397A^*TLA* plasmid | This study |
| CgH-I397V | LCgL1 derivative harboring pEC-*H*^I397V^*TLA* plasmid | This study |
| CgH-DM | LCgL1 derivative harboring pEC-*H*^A384D+397V^*TLA* plasmid | This study |
| E1(WT) | Transetta (DE3) derivative harboring pET-*hom* plasmid | This study |
| E1(G378E) | Transetta (DE3) derivative harboring pET-*hom*^G378E^ plasmid | This study |
| E1(A384D) | Transetta (DE3) derivative harboring pET-*hom*^A384D^ plasmid | This study |
| E1(I397V) | Transetta (DE3) derivative harboring pET-*hom*^I397V^ plasmid | This study |
| E1(28a) | Transetta (DE3) derivative harboring pET-28a plasmid | This study |

a ATCC: American Type Culture Collection.

**Supplementary Table 2.** Plasmids used in this study

| **Plasmid** | **Description^a^** | **Source** |
| --- | --- | --- |
| pCas9gRNA-*ccdB* | Temperature-sensitive plasmid for genetic edition in *C. glutamicum*, Cm^R^ | (Liu et al., 2022) |
| pEC-XK99E | *E. coli*-*C. glutamicum* shuttle expression vector; *P_trc_*, Km^R^ | (Kirchner and Tauch, 2003) |
| pCas9-gRNA-Δ*hom* | pCas9gRNA-*ccdB* derivative carrying gRNA target *hom* and homology arms for deletion of *hom* | This study |
| pEC-*homthrB* | pEC-XK99E derivative carrying a *hom-thrB* operon of ATCC13032 | This study |
| pEC-*hom*^G378E^*thrB* | pEC-*homthrB* derivative with G378E mutation of *hom* | This study |
| pEC*-HTLA* | pEC-*homthrB* derivative carrying a *lysC*^I293Y^*-asd* operon driven by *P*_pyc_-13 promoter | This study |
| pEC*-H*^G378E^*TLA* | pEC*-HTLA* derivative with G378E mutation of *hom* | This study |
| pEC-*H*^A381V^*TLA* | pEC*-HTLA* derivative with A381V mutation of *hom* | This study |
| pEC-*H*^A381P^*TLA* | pEC*-HTLA* derivative with A381P mutation of *hom* | This study |
| pEC-*H*^A384D^*TLA* | pEC*-HTLA* derivative with A384D mutation of *hom* | This study |
| pEC-*H*^I397R^*TLA* | pEC*-HTLA* derivative with I397R mutation of *hom* | This study |
| pEC-*H*^I397V^*TLA* | pEC*-HTLA* derivative with I397V mutation of *hom* | This study |
| pEC-*H*^I397A^*TLA* | pEC*-HTLA* derivative with I397A mutation of *hom* | This study |
| pEC-*H*^A384D+I397V^*TLA* | pEC*-HTLA* derivative with A384D and I397V mutations of *hom* | This study |
| pET-*hom* | pET-28a derivative carrying a *hom* cassette of ATCC13032 | This study |
| pET-*hom*^G378E^ | pET-*hom* derivative with G378E mutation of *hom* | This study |
| pET-*hom*^A384D^ | pET-*hom* derivative with A384D mutation of *hom* | This study |
| pET-*hom*^I397V^ | pET-*hom* derivative with I397V mutation of *hom* | This study |

^a^Km^R^ and Cm^R^ represent resistance to kanamycin and chloramphenicol, respectively.

**Supplementary Table 3.** Primers used in this study.

| **Plasmid** | **Primer** | **Sequence 5’-3’** | **PCR template** | **Plasmid construction process^a^** |
| --- | --- | --- | --- | --- |
| pCas9-gRNA-*Δhom* | Cas9-1 | tcgaagggcaccaataactgc | pCas9gRNA-*ccdB* | Ligation of five PCR products via recombination |
|  | Cas9-2 | cttttactttcaccagcgtttctg |  |  |
|  | Cas9-3 | aacgctggtgaaagtaaaagatgc | pCas9gRNA-*ccdB* |  |
|  | Cas9-4 | TCGCTTTCCACACCCGTGTTtgaattacactgtacctgttgcgtc |  |  |
|  | gRNA-1 | AACACGGGTGTGGAAAGCGAgttttagagctagaaatag | pCas9gRNA-*ccdB* |  |
|  | gRNA-2 | caacctgccatcacgagattttc |  |  |
|  | *hom*-1 | aatctcgtgatggcaggttgCACAATTTCTTTGCCCAGTTCG | Genomic DNA of *C. glutamicum* |  |
|  | *hom*-2 | GCATCATCATCGCGCTCTTCGTACTCGGTCATCAGACGCATC |  |  |
|  | *hom*-3 | GAAGAGCGCGATGATGATGC | Genomic DNA of *C. glutamicum* |  |
|  | *hom*-4 | cagttattggtgcccttcgaGAAGCCGCAGCATTATCTGG |  |  |
| pEC-*homthrB* | PEC-F | ctgcaggcatgcaagcttgg | pEC-8 (Liu et al., 2022) | Ligation of two PCR products via recombination |
|  | PEC-R | TCTCAACTCCTTTGGCCTGTGTGAAC |  |  |
|  | *homthrB*-F | ACAGGCCAAAGGAGTTGAGAATGACCTCAGCATCTGCCCCAAG | Genomic DNA of *C. glutamicum* |  |
|  | *homthrB*-R | ccaagcttgcatgcctgcagCTAAGGTTGGTTAACTTCAACCTTGACTGGTCC |  |  |
| pEC-*hom*^G378E^*thrB* | PEC-F | ctgcaggcatgcaagcttgg | pEC-*homthrB* | Ligation of three PCR products via recombination |
|  | PEC-R | TCTCAACTCCTTTGGCCTGTGTGAAC |  |  |
|  | *homthrB*-F | ACAGGCCAAAGGAGTTGAGAATGACCTCAGCATCTGCCCCAAG | pEC-*homthrB* |  |
|  | 378-R | CTCCACGCGATCTTCCACATC |  |  |
|  | 378E-F | GATGTGGAAGATCGCGTGGAGGTTTTGGCTGAATTG | pEC-*homthrB* |  |
|  | *homthrB*-R | ccaagcttgcatgcctgcagCTAAGGTTGGTTAACTTCAACCTTGACTGGTCC |  |  |
| pEC-*hom*^375^*thrB*(library) | 375-F | CACCTCGACATGGATGTGGAANNNCGCGTGGGGGTTTTGGCTG | pEC-*homthrB* | Ligation of two PCR products via recombination |
|  | PEC-2 | CATCCGCCAAAACAGCCAAG |  |  |
|  | PEC-1 | cttggctgttttggcggatgagag | pEC-*homthrB* |  |
|  | 375-R | CCACATCCATGTCGAGGTGG |  |  |
| pEC-*hom*^380^*thrB*(library) | 380-F | GAAGATCGCGTGGGGGTTNNNGCTGAATTGGCTAGCCTGTTCTC | pEC-*homthrB* | Ligation of two PCR products via recombination |
|  | PEC-2 | CATCCGCCAAAACAGCCAAG |  |  |
|  | PEC-1 | cttggctgttttggcggatgagag | pEC-*homthrB* |  |
|  | 380-R | AACCCCCACGCGATCTTC |  |  |
| pEC-*hom*^381^*thrB*(library) | 381-F | AAGATCGCGTGGGGGTTTTGNNNGAATTGGCTAGCCTGTTCTC | pEC-*homthrB* | Ligation of two PCR products via recombination |
|  | PEC-2 | CATCCGCCAAAACAGCCAAG |  |  |
|  | PEC-1 | cttggctgttttggcggatgagag | pEC-*homthrB* |  |
|  | 381-R | CAAAACCCCCACGCGATCTTC |  |  |
| pEC-*hom*^384^*thrB*(library) | 384-F | GGGGGTTTTGGCTGAATTGNNNAGCCTGTTCTCTGAGCAAGG | pEC-*homthrB* | Ligation of two PCR products via recombination |
|  | PEC-2 | CATCCGCCAAAACAGCCAAG |  |  |
|  | PEC-1 | cttggctgttttggcggatgagag | pEC-*homthrB* |  |
|  | 384-R | CAATTCAGCCAAAACCCCC |  |  |
| pEC-*hom*^392^*thrB*(library) | 392-F | GCCTGTTCTCTGAGCAAGGANNNTCCCTGCGTACAATCCGACAGGAAG | pEC-*homthrB* | Ligation of two PCR products via recombination |
|  | PEC-2 | CATCCGCCAAAACAGCCAAG |  |  |
|  | PEC-1 | cttggctgttttggcggatgagag | pEC-*homthrB* |  |
|  | 392-R | TCCTTGCTCAGAGAACAGGC |  |  |
| pEC-*hom*^393^*thrB*(library) | 393-F | CTGTTCTCTGAGCAAGGAATCNNNCTGCGTACAATCCGACAGG | pEC-*homthrB* | Ligation of two PCR products via recombination |
|  | PEC-2 | CATCCGCCAAAACAGCCAAG |  |  |
|  | PEC-1 | cttggctgttttggcggatgagag | pEC-*homthrB* |  |
|  | 393-R | ATTCCTTGCTCAGAGAACAGGCTAG |  |  |
| pEC-*hom*^394^*thrB*(library) | 394-F | GTTCTCTGAGCAAGGAATCTCCNNNCGTACAATCCGACAGGAAGAGC | pEC-*homthrB* | Ligation of two PCR products via recombination |
|  | PEC-2 | CATCCGCCAAAACAGCCAAG |  |  |
|  | PEC-1 | cttggctgttttggcggatgagag | pEC-*homthrB* |  |
|  | 394-R | GATTCCTTGCTCAGAGAACAGGCTAG |  |  |
| pEC-*hom*^397^*thrB*(library) | 397-F | CAAGGAATCTCCCTGCGTACANNNCGACAGGAAGAGCGCG | pEC-*homthrB* | Ligation of two PCR products via recombination |
|  | PEC-2 | CATCCGCCAAAACAGCCAAG |  |  |
|  | PEC-1 | cttggctgttttggcggatgagag | pEC-*homthrB* |  |
|  | 397-R | GTACGCAGGGAGATTCCTTGC |  |  |
| pEC-*hom*^399^*thrB*(library) | 399-F | CTCCCTGCGTACAATCCGANNNGAAGAGCGCGATGATGATGC | pEC-*homthrB* | Ligation of two PCR products via recombination |
|  | PEC-2 | CATCCGCCAAAACAGCCAAG |  |  |
|  | PEC-1 | cttggctgttttggcggatgagag | pEC-*homthrB* |  |
|  | 399-R | TCGGATTGTACGCAGGGAGATTC |  |  |
| pEC-*HTLA* | P*_pyc_*-F | gttgaagttaaccaaccttaggaaaacccaggattgctttg | *P_pyc_*_-13_ variant plasmid (Liu et al., 2022) | Ligation of three PCR products via recombination |
|  | P*_pyc_*-R | TAGAGTAATTATTCCTTTCAACAAGAGACC |  |  |
|  | LA-F | TGAAAGGAATAATTACTCTAGTGGCCCTGGTCGTACAGAAATATG | Genomic DNA of ZCgLJ6 |  |
|  | LA-R | ctgagctgctggttaagtaactgcaggcatgcaagcttgg |  |  |
|  | pEC1-F | ctgcaggcatgcaagcttggctg | pEC-*homthrB* |  |
|  | pEC1-R | ctaaggttggttaacttcaac |  |  |
| pEC-*H*^G378E^*TLA* | Bone-F | gttgaagttaaccaaccttag | pEC-*HTLA* | Ligation of three PCR products via recombination |
|  | Bone-R | gaacatgtgagcaaaaggcc |  |  |
|  | HT-F | ggccttttgctcacatgttctttcctgcgttatcccctg | pEC-*HTLA* |  |
|  | 378-R | CTCCACGCGATCTTCCACATC |  |  |
|  | 378E-F | GATGTGGAAGATCGCGTGGAGGTTTTGGCTGAATTG | pEC-*HTLA* |  |
|  | HT-R | ctaaggttggttaacttcaaccttgactggtcccgcaacc |  |  |
| pEC-*H*^A381V^*TLA* | Bone-F | gttgaagttaaccaaccttag | pEC-*HTLA* | Ligation of three PCR products via recombination |
|  | Bone-R | gaacatgtgagcaaaaggcc |  |  |
|  | HT-F | ggccttttgctcacatgttctttcctgcgttatcccctg | pEC-*HTLA* |  |
|  | 381-R | CAAAACCCCCACGCGATCTTC |  |  |
|  | 381V-F | AAGATCGCGTGGGGGTTTTGGTTGAATTGGCTAGCCTGTTCTC | pEC-*HTLA* |  |
|  | HT-R | ctaaggttggttaacttcaaccttgactggtcccgcaacc |  |  |
| pEC-*H*^A381P^*TLA* | Bone-F | gttgaagttaaccaaccttag | pEC-*HTLA* | Ligation of three PCR products via recombination |
|  | Bone-R | gaacatgtgagcaaaaggcc |  |  |
|  | HT-F | ggccttttgctcacatgttctttcctgcgttatcccctg | pEC-*HTLA* |  |
|  | 381-R | CAAAACCCCCACGCGATCTTC |  |  |
|  | 381P-F | AAGATCGCGTGGGGGTTTTGCCGGAATTGGCTAGCCTGTTCTC | pEC-*hom*^A381P^*thrB* |  |
|  | HT-R | ctaaggttggttaacttcaaccttgactggtcccgcaacc |  |  |
| pEC-*H*^A384D^*TLA* | Bone-F | gttgaagttaaccaaccttag | pEC-*HTLA* | Ligation of three PCR products via recombination |
|  | Bone-R | gaacatgtgagcaaaaggcc |  |  |
|  | HT-F | ggccttttgctcacatgttctttcctgcgttatcccctg | pEC-*HTLA* |  |
|  | 384-R | CAATTCAGCCAAAACCCCC |  |  |
|  | 384D-F | GGGGGTTTTGGCTGAATTGGATAGCCTGTTCTCTGAGCAAGG | pEC-*HTLA* |  |
|  | HT-R | ctaaggttggttaacttcaaccttgactggtcccgcaacc |  |  |
| pEC-*H*^I397R^*TLA* | Bone-F | gttgaagttaaccaaccttag | pEC-*HTLA* | Ligation of three PCR products via recombination |
|  | Bone-R | gaacatgtgagcaaaaggcc |  |  |
|  | HT-F | ggccttttgctcacatgttctttcctgcgttatcccctg | pEC-*HTLA* |  |
|  | 397-R | GTACGCAGGGAGATTCCTTGC |  |  |
|  | 397R-F | CAAGGAATCTCCCTGCGTACAAGGCGACAGGAAGAGCGCG | pEC-*HTLA* |  |
|  | HT-R | ctaaggttggttaacttcaaccttgactggtcccgcaacc |  |  |
| pEC-*H*^I397A^*TLA* | Bone-F | gttgaagttaaccaaccttag | pEC-*HTLA* | Ligation of three PCR products via recombination |
|  | Bone-R | gaacatgtgagcaaaaggcc |  |  |
|  | HT-F | ggccttttgctcacatgttctttcctgcgttatcccctg | pEC-*HTLA* |  |
|  | 397-R | GTACGCAGGGAGATTCCTTGC |  |  |
|  | 397A-F | CAAGGAATCTCCCTGCGTACAGCCCGACAGGAAGAGCGCG | pEC-*HTLA* |  |
|  | HT-R | ctaaggttggttaacttcaaccttgactggtcccgcaacc |  |  |
| pEC-*H*^I97V^*TLA* | Bone-F | gttgaagttaaccaaccttag | pEC-*HTLA* | Ligation of three PCR products via recombination |
|  | Bone-R | gaacatgtgagcaaaaggcc |  |  |
|  | HT-F | ggccttttgctcacatgttctttcctgcgttatcccctg | pEC-*HTLA* |  |
|  | 397-R | GTACGCAGGGAGATTCCTTGC |  |  |
|  | 397V-F | GAAGATCGCGTGGGGGTTTTGGTGGAATTGGCTAGCCTGTTCTC | pEC-*HTLA* |  |
|  | HT-R | ctaaggttggttaacttcaaccttgactggtcccgcaacc |  |  |
| pEC-*H*^A384D+I397V^*TLA* | Bone-F | gttgaagttaaccaaccttag | pEC-*HTLA* | Ligation of three PCR products via recombination |
|  | Bone-R | gaacatgtgagcaaaaggcc |  |  |
|  | HT-F | ggccttttgctcacatgttctttcctgcgttatcccctg | pEC-*H*^A384D^*TLA* |  |
|  | 397-R | GTACGCAGGGAGATTCCTTGC |  |  |
|  | 397V-F | CAAGGAATCTCCCTGCGTACAGTGCGACAGGAAGAGCGCG | pEC-*HTLA* |  |
|  | HT-R | ctaaggttggttaacttcaaccttgactggtcccgcaacc |  |  |
| pET-*hom* | 28a-F | atggctgccgcgcggcaccag | pET-28a | Ligation of two PCR products via recombination |
|  | 28a-R | caaagcccgaaaggaagc |  |  |
|  | *hom*-F | gcttcctttcgggctttgTTAGTCCCTTTCGAGGCGGATC | Genomic DNA of *C. glutamicum* |  |
|  | *hom*-R | ctggtgccgcgcggcagccatATGACCTCAGCATCTGCCCC |  |  |
| pET-*hom*^G378E^ | 28a-F | atggctgccgcgcggcaccag | pET-28a | Ligation of two PCR products via recombination |
|  | 28a-R | caaagcccgaaaggaagc |  |  |
|  | *hom*-F | gcttcctttcgggctttgTTAGTCCCTTTCGAGGCGGATC | pEC*-H*^G378E^*TLA* |  |
|  | *hom*-R | ctggtgccgcgcggcagccatATGACCTCAGCATCTGCCCC |  |  |
| pET-*hom*^A384D^ | 28a-F | atggctgccgcgcggcaccag | pET-28a | Ligation of two PCR products via recombination |
|  | 28a-R | caaagcccgaaaggaagc |  |  |
|  | *hom*-F | gcttcctttcgggctttgTTAGTCCCTTTCGAGGCGGATC | pEC*-H*^A384D^*TLA* |  |
|  | *hom*-R | ctggtgccgcgcggcagccatATGACCTCAGCATCTGCCCC |  |  |
| pET-*hom*^I397V^ | 28a-F | atggctgccgcgcggcaccag | pET-28a | Ligation of two PCR products via recombination |
|  | 28a-R | caaagcccgaaaggaagc |  |  |
|  | *hom*-F | gcttcctttcgggctttgTTAGTCCCTTTCGAGGCGGATC | pEC*-H*^I397V^*TLA* |  |
|  | *hom*-R | ctggtgccgcgcggcagccatATGACCTCAGCATCTGCCCC |  |  |

**Supplementary Table 4.** Mutants from AHV-resistant plate screening.

| **Libraries** | **Degenerate codon** | **amino acid substitution** | **Resistant variants** |
| --- | --- | --- | --- |
| 381 | GCT→GTT | A381V | CgL- A381V1 |
|  | GCT→GTA | A381V | CgL- A381V2 |
|  | GCT→GTG | A381V | CgL- A381V3 |
|  | GCT→GAT | A381D | CgL- A381D |
|  | GCT→ATA | A381I | CgL- A381I1 |
|  | GCT→ATC | A381I | CgL- A381I2 |
|  | GCT→ATT | A381I | CgL- A381I3 |
|  | GCT→TTA | A381L | CgL- A381L |
|  | GCT→CCG | A381P | CgL- A381P1 |
|  | GCT→CCT | A381P | CgL- A381P2 |
| 384 | GCT→CGT | A384R | CgL- A384R |
|  | GCT→GAT | A384D | CgL- A384D |
| 397 | ATC→TGC | I397C | CgL- I397C |
|  | ATC→GAT | I397D | CgL- I397D |
|  | ATC→GGC | I397G | CgL- I397G |
|  | ATC→AGC | I397S | CgL- I397S1 |
|  | ATC→AGT | I397S | CgL- I397S2 |
|  | ATC→AGG | I397R | CgL- I397R1 |
|  | ATC→CGT | I397R | CgL- I397R2 |
|  | ATC→GTG | I397V | CgL- I397V1 |
|  | ATC→GTC | I397V | CgL- I397V2 |
|  | ATC→GTT | I397V | CgL- I397V3 |
|  | ATC→GCC | I397A | CgL- I397A |

**Supplementary Table 5.** Analysis of the changes in l-threonine binding free energy (ΔΔG_­­_^Thr^_binding_).

| **mutants** | **ΔΔG_­­_^Thr^_binding_** | **Effect of Mutation** |
| --- | --- | --- |
| G378E | 2.66 | destabilizing |
| I397V | 2.94 | destabilizing |
| A384D | 2.89 | destabilizing |

# References

Dong, X., Zhao, Y., Zhao, J., and Wang, X. (2016). Characterization of aspartate kinase and homoserine dehydrogenase from *Corynebacterium glutamicum* IWJ001 and systematic investigation of l-isoleucine biosynthesis. *J Ind Microbiol Biotechnol* 43 (6), 873-885. doi: 10.1007/s10295-016-1763-5

Kirchner, O., and Tauch, A. (2003). Tools for genetic engineering in the amino acid-producing bacterium *Corynebacterium glutamicum*. *J. Biotechnol.* 104 (1-3), 287-299. doi: 10.1016/s0168-1656(03)00148-2

Liu, J., Liu, M., Shi, T., Sun, G., Gao, N., Zhao, X., et al. (2022). CRISPR-assisted rational flux-tuning and arrayed CRISPRi screening of an l-proline exporter for l-proline hyperproduction. *Nat. Commun.* 13 (1), 891. doi: 10.1038/s41467-022-28501-7
